# Carotenoids of Lettuce (*Lactuca sativa* L.) Grown on Soil Enriched with Spent Coffee Grounds

**DOI:** 10.3390/molecules17021535

**Published:** 2012-02-07

**Authors:** Rebeca Cruz, Paula Baptista, Sara Cunha, José Alberto Pereira, Susana Casal

**Affiliations:** 1 REQUIMTE, Laboratory of Bromatology and Hydrology, Faculty of Pharmacy, Porto University, Rua de Jorge Viterbo Ferreira 228, 4050-313 Porto, Portugal; Email: rebecca.ccruz@gmail.com (R.C.); sara.cunha@ff.up.pt (S.C.); 2 Mountain Research Centre (CIMO), School of Agriculture, Polytechnic Institute of Bragança, Campus Santa Apolónia, Apartado 1172, Bragança 5301-855, Portugal; Email: pbaptista@ipb.pt (P.B.); jpereira@ipb.pt (J.A.P.)

**Keywords:** β-carotene, lutein, chlorophyll, spent coffee grounds, soil amendment, lettuce

## Abstract

The impact of spent coffee grounds on carotenoid and chlorophyll content in lettuce (*Lactuca sativa* L. var. *capitata*) was evaluated. A greenhouse pot experiment was conducted with spent coffee amounts ranging from 0% to 20% (v/v). All evaluated pigments increased proportionally to spent coffee amounts. Lutein and β-carotene levels increased up to 90% and 72%, respectively, while chlorophylls increased up to 61%. Biomass was also improved in the presence of 2.5% to 10% spent coffee, decreasing for higher amounts. Nevertheless, all plants were characterized by lower organic nitrogen content than the control ones, inversely to the spent coffee amounts, pointing to possible induced stress. Collected data suggests that plants nutritional features, with regards to these bioactive compounds, can be improved by the presence of low amounts of spent coffee grounds (up to 10%). This observation is particularly important because soil amendment with spent coffee grounds is becoming increasingly common within domestic agriculture. Still, further studies on the detailed influence of spent coffee bioactive compounds are mandatory, particularly regarding caffeine.

## 1. Introduction

Coffee is one of the most popular beverages worldwide. The coffee processing chain, however, leads to the accumulation of diverse organic residues. Major solid wastes in the producing countries comprise coffee husks and pulps, depending on the green processing method applied, and correspond to more than 80% of the crop (fresh basis). In the consuming countries, the residues are almost limited to spent coffee grounds (SCG) obtained after the brewing process in cafeterias and restaurants, at home, or by the soluble coffee industry. Estimated worldwide annual production of SCG is around 6 million tons [1]. As with other agricultural and food wastes, coffee solid residues have high volume but low value, which results in reduced global economic interest. Thus, generally, they are used either for energy production or disposed to landfill, causing major environmental issues. Several studies have been published on the last decade dealing with possible strategies to reduce their toxicity and enhance their commercial value, including animal feed, mushroom production, biodiesel, fuel pellets or activated carbons [1,2].

The excessive use of organic fertilizers in agriculture is also an issue of global concern, due to the associated decrease in soil fertility, increase bulk density, soil and ground water pollution, and contamination of produced vegetables, all with direct consequences on human health. Simultaneously, consumers are increasingly demanding healthier foods, in a global world where healthfulness is associated with the amounts of bioactive compounds, including vitamins, carotenoids, phenolic compounds, *etc*. Agricultural and food wastes are regarded as good alternatives to chemical fertilizers, allowing recovery of the soil structure and maintenance of its fertility. SCG could be such an interesting approach: their rigid cellular structure can contribute to the recovery of soils structure and aeration [3], while its residual organic compounds can enhance the organic content, particularly N, and therefore fertility. Indeed, and despite the absence of scientific evidence upon its effectiveness, or even its safety, the use of SCG in domestic agriculture is an increasing popular practice. If we take a deep look into the chemical composition of SCG, their bioactive compounds, such as caffeine and chlorogenic acids residual amounts, might present some toxicity to the soil microorganisms, decreasing soil capability to release N. Also, caffeine uptake by plants, as already observed in mushrooms (*Pleurotus* sp.) by Fan and co-workers [4] must be accounted for, with potential implication on physiology and nutritional status. Nevertheless, caffeine can also be regarded as a natural pesticide, reducing the need for further chemicals, and complying with the increasing demand for organic agriculture practices.

The work presented herein is part of a project aiming to verify the feasibility of using coffee grounds for agricultural purposes, particularly regarding the nutritional quality of the vegetables obtained and its consequent environmental impact. Since carotenoids and chlorophylls are sensitive to plant growth conditions [5,6] it is of great interest to evaluate the effect of SCG upon their amounts. For the purpose of this work, the detailed study of these plant metabolites can give indications of the plant physiological status and visual quality attributes [6]. Carotenoids are located in the subcellular organelles, like chloroplasts, were they serve as light harvesting pigments for photosynthesis [7]. Simultaneously, carotenoids are potent antioxidants, being important for photoprotection of the photosynthetic components [8] and among the most important bioactive compounds obtained from vegetable consumption [9,10]. Besides the well-known provitamin A activity of some of these compounds, their antioxidant activity has also been associated with lowered risk of developing degenerative diseases such as cancer, cardiovascular diseases, cataract and macular degeneration [11]. On the other hand, chlorophylls are also important plant pigments in green leafy vegetables and are increasingly linked to some of the positive effects associated with vegetable consumption, particularly its potential cancer preventive activity [12]. Their collective study might give further insight into the effect of SCG on green leafy vegetables from both the physiological and nutritional points of view.

Lettuce was selected for the present study. It is cultivated worldwide, and is one the most consumed green leafy vegetables in the raw form for its taste and high nutritive value, being regarded as an important source of phytonutrients, including carotenoids, in the diet [9,10]. *Lactuca sativa* L. var. *capitata* cv. “Four Seasons” was selected for the present study. As with other plants, the amount of bioactive substances is known to depend on several parameters, including cultivar characteristics, climate conditions, seasonal changes, maturity, post-harvest treatment and soil characteristics. This last parameter was also tested by using different amounts of SCG addition to the soil used for its cultivation.

## 2. Results and Discussion

### 2.1. Effect of SCG on Carotenoid and Chlorophyll Content in Lettuce Leaves

**Figure 1:**
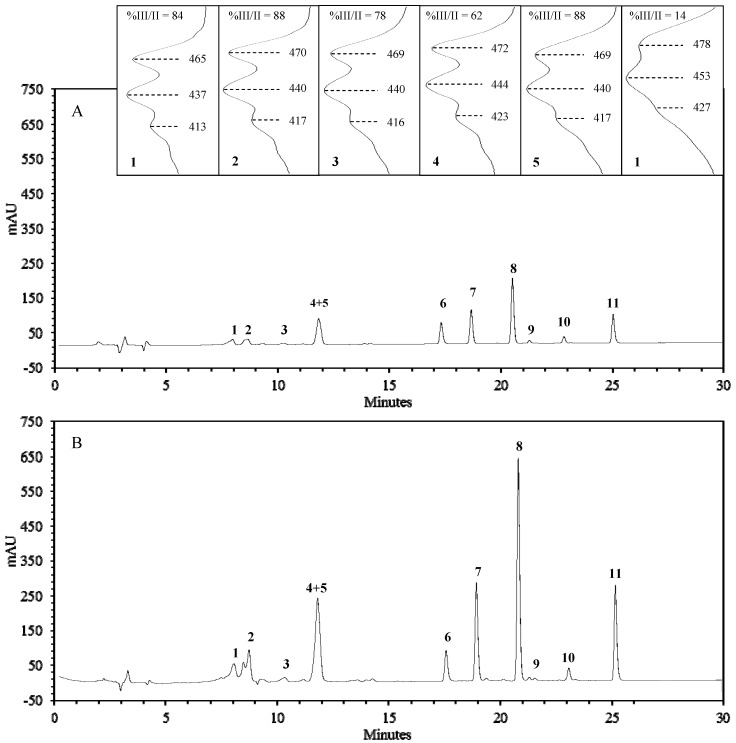
Representative chromatograms of lettuce extracts from a control group (**A**) and a 15% SCG group (**B**), recorded at 440 nm. (**1**) Neoxanthin; (**2**) Violaxanthin; (**3**) Lactucaxanthin; (**4**) Lutein; (**5**) Lutein-5,6-epoxide; (**6**) *trans*-β-apo-8′-carotenal (internal standard); (**7**) Chlorophyll *b*; (**8**) Chlorophyll *a*; (**9**) Pheophytin *b*; (**10**) Pheophytin *a*; (**11**) β-Carotene.

Most reports on green leaf carotenoids and chlorophylls are based on direct spectrophotometric measurements of sample extracts. For the present work, a detailed carotenoid and chlorophyll composition was obtained by use of HPLC analysis, allowing a better understanding of the SCG influence on these pigments. Several “carotenoid-like compounds” were detected in the sample extracts but, on the basis of their chromatographic resolution, spectral characteristics [13], amounts, and literature data for similar matrices, only five were quantified. The two major ones (lutein and β-carotene) are detailed individually (Table 1), while the others (neoxanthin, violaxanthin and lactucaxanthin) are included under the collective designation of total carotenoids (Table 2). Co-elution of lutein with a “carotenoid-like” compound with spectral characteristics similar to lutein-5,6-epoxide (Figure 1) was observed. Therefore, when discussing lutein amounts, “lutein plus epoxide” should be assumed. Regarding chlorophylls, both chlorophyll *a* (Chl A) and *b* (Chl B), and their related pheophytins when present, were quantified in all samples.

**Table 1:** Detail on individual carotenoids and chlorophylls amounts in lettuce grown with SCG.

% SCG	Group	Lutein	β-Carotene	Chlorophyll *a*	Chlorophyll *b*
		mg/100 g	mg/100 g	mg/100 g	mg/100 g
**0**	1	3.77 ± 0.03	3.51 ± 0.33	12.70 ± 0.06	3.44 ± 0.85
	2	3.06 ± 0.10	2.27 ± 0.07	8.04 ± 0.10	3.42 ± 1.10
	3	4.21 ± 0.70	3.52 ± 0.57	13.00 ± 1.74	4.98 ± 0.55
	4	3.87 ± 0.55	3.31 ± 0.44	12.61 ± 1.80	4.11 ± 0.46
	5	4.49 ± 0.18	3.84 ± 0.30	11.62 ± 1.03	4.03 ± 0.10
	**n = 15**	**3.92 ± 0.65 ^a^**	**3.33 ± 0.66 ^a^**	**11.94 ± 2.06 ^a^**	**4.00 ± 0.79 ^a^**
**2.5**	1	4.85 ± 0.90	4.24 ± 0.30	13.04 ± 0.06	5.06 ± 0.62
	2	4.01 ± 0.03	4.84 ± 0.96	12.79 ± 0.03	5.68 ± 2.19
	3	4.16 ± 0.56	3.55 ± 0.39	13.40 ± 1.10	4.85 ± 0.92
	4	6.92 ± 0.00	4.85 ± 0.18	11.02 ± 0.09	4.10 ± 0.28
	5	4.48 ± 0.86	3.57 ± 0.37	13.71 ± 0.06	4.21 ± 1.46
	**n = 15**	**4.55 ± 0.92 ^a^**	**4.21 ± 0.79 ^a^**	**12.96 ± 1.11 ^a,b^**	**4.78 ± 1.13 ^a,b^**
**5**	1	6.72 ± 0.42	4.55 ± 0.27	15.61 ± 1.15	6.11 ± 0.48
	2	7.54 ± 0.00	4.82 ± 1.13	13.80 ± 2.99	5.65 ± 1.09
	3	6.39 ± 0.61	4.30 ± 0.32	14.71 ± 1.34	4.68 ± 1.13
	4	5.74 ± 0.26	4.03 ± 0.26	13.95 ± 0.73	4.75 ± 0.66
	**n = 12**	**6.41 ± 0.69 ^b^**	**4.42 ± 0.64 ^b^**	**14.58 ± 1.64 ^b,c^**	**5.41 ± 0.95 ^a,b^**
**10**	1	7.38 ± 0.90	4.97 ± 0.53	16.96 ± 1.93	6.96 ± 0.41
	2	8.43 ± 0.45	5.54 ± 0.22	18.80 ± 2.09	5.94 ± 1.83
	3	7.13 ± 0.04	4.38 ± 0.96	14.82 ± 3.64	5.38 ± 0.30
	4	5.20 ± 0.30	3.57 ± 0.45	12.48 ± 0.91	5.00 ± 0.10
	5	5.51 ± 0.00	4.57 ± 0.51	15.50 ± 2.12	4.94 ± 1.18
	**n= 15**	**6.83 ± 1.34 ^b,c^**	**4.55 ± 0.84 ^b^**	**15.84 ± 3.05^ c,d^**	**5.64 ± 1.11 ^a,b^**
**15**	1	8.58 ± 0.23	6.83 ± 1.14	17.01 ± 3.83	10.51 ± 2.43
	2	7.21 ± 0.77	6.23 ± 1.36	17.68 ± 1.19	7.93 ± 1.88
	3	7.34 ± 0.36	4.96 ± 0.94	18.79 ± 0.01	7.22 ± 1.38
	4	7.02 ± 0.04	5.31 ± 0.06	17.79 ± 0.03	6.63 ± 0.21
	5	7.42 ± 0.64	4.74 ± 0.37	15.24 ± 1.56	6.26 ± 1.33
	**n = 15**	**7.44 ± 0.67 ^c^**	**5.70 ± 1.23 ^c^**	**17.20 ± 1.87^ d^**	**7.96 ± 2.19 ^c^**
**20**	1	6.49 ± 0.02	5.05 ± 0.01	18.96 ± 0.03	5.23 ± 1.82
	2	4.64 ± 0.01	3.80 ± 0.25	11.32 ± 0.00	6.05 ± 1.25
	3	6.66 ± 1.27	4.96 ± 1.35	17.57 ± 3.62	6.44 ± 1.46
	4	5.96 ± 0.54	4.66 ± 0.74	14.75 ± 0.04	6.27 ± 2.19
	5	8.18 ± 0.01	5.17 ± 0.41	19.67 ± 0.05	5.17 ± 0.31
	6	8.08 ± 1.29	6.07 ± 0.59	19.88 ± 0.00	5.55 ± 2.81
	**n =18**	**6.61 ± 1.35 ^b,c^**	**4.95 ± 0.92 ^b,c^**	**17.33 ± 3.14 ^d^**	**5.79 ± 1.44^ b^**

All results are presented as mg/100 g of fresh weight lettuce leaves. ^a–d^ indicate the SCG treatments that have statistically significant differences (*p* < 0.05) from the given mean. Analytical details in experimental section.

Figure 1 represents a chromatogram of a control lettuce group (A) and a lettuce group grown with 15% (v/v) of spent coffee (B), with detailed spectra for major compounds. All samples showed a similar carotenoid profile, with higher amounts of lutein than β-carotene, and reduced amounts of neoxanthin, violaxanthin and lactucaxanthin. Chl A was always higher than Chl B.

Table 1 details the amounts of lutein, β-carotene, Chl A and B for each SCG group. All SCG percentages are represented by four to six sample groups, each one representing a composite sample of three lettuce plants. The individual standard deviation was obtained from triplicate extractions. Under the present study the maturity at harvest, climate/season/geographic site of production, part of the plant utilized, agricultural practices, harvesting and post-harvest handling were identical to all samples. The differences observed are, thus, directly related to the composition of cultivation medium, the objective of this study.

In lettuce control groups (Table 1), β-carotene ranged from 2.27 mg/100 g to 3.84 mg/100 g, on a fresh basis. These amounts are in accordance with the literature, namely with Cardoso and co-workers [14], who reported values ranging from 2.82 mg/100 g to 3.58 mg/100 g during spring season, or with Kim and co-workers [15] with 2 mg/g. Lutein ranged from 3.06 mg/100 g to 4.49 mg/100 g, also in accordance with this previous author. Mou [16] reported slightly lower amounts but highlighted the high variability shown among lettuce varieties and cultivars. Chl A was detected in higher amounts than Chl B, with 11.94 mg/100 g in the former and 4.00 mg/100 g in the latter. All the reported amounts are in accordance with the literature [17].

The carotenoid concentrations in lettuce were signiﬁcantly affected by the presence of SCG in cultivation medium. Regardless of the analysed carotenoid, there is a noteworthy improvement with the increase of SCG in cultivation medium, for all tested amounts. Lutein ranged from 4.01 mg/100 g to 8.58 mg/100 g, while β-carotene varied from 3.55 mg/100 g to 6.83 mg/100 g. All results are present on a fresh basis, as consumed. Except for the 2.5% groups, all the other treatments reveal statistically higher (*p* < 0.05) lutein amounts than the control groups. A 90% lutein raise was observed in the 15% group, without statistical differences from the 10% and 20% groups, both with increments around 70% (Table 1). For β-carotene, no significant differences were observed between the 2.5%, 5% and 10% groups, but all three presented significantly higher amounts than control. β-Carotene content was again higher for the 15% SCG group, showing a 72% increment relatively to the control. Both chlorophylls (Table 1) evidenced a similar pattern, with an almost linear increase up to the 15% group.

Based on the previous observations, one might infer that the increase in carotenoids and chlorophylls can be regarded as favourable towards the nutritional benefits of these products. Being represented on a fresh basis, direct estimations on the pigment amounts in lettuce portion can be easily performed. Nevertheless, this effect cannot be discussed alone, as other parameters, such as plant physiological status and biomass production, are equally or even important.

### 2.2. Correlation with Other Parameters Evaluated

The edible part of each lettuce plant was weighted before being grouped and homogenized for the chemical analyses. The results are reported on Table 2. Particularly noticeable is the high variability in sample edible biomass. Nevertheless, an increment of fresh leaves medium weight occurred from control to 2.5% and 5% samples, reaching a 25% increase in the latter, when compared to the control. For higher SCG amounts, the biomass was reduced to values inferior to those observed in control samples.

**Table 2:** Leaves biomass, organic nitrogen, and total pigments as function of SCG amounts.

% SCG	Leaves biomass	Organic nitrogen	Total carotenoids	Total chlorophylls
	(g)	(g/100g DW)	(mg/100g, FW)	(mg/100g, FW)
**0**	22.13 ± 7.11 ^a,b^	3.84 ± 0.25 ^a^	9.74 ± 1.15 ^a^	15.59 ± 2.47 ^a^
**2.5**	21.71 ± 4.06 ^a,b^	3.60 ± 0.02 ^a,b,c^	12.51 ± 1.44 ^a^	17.57 ± 1.38 ^a,b^
**5**	27.56 ± 6.40 ^a^	3.34 ± 0.11 ^b^	17.01 ± 1.42 ^b^	19.82 ± 1.31 ^a,b,c^
**10**	17.28 ± 6.36 ^b^	3.67 ± 0.21 ^c^	17.86 ± 3.05 ^b^	21.36 ± 2.97 ^b,c^
**15**	9.65 ± 4.64 ^c^	2.87 ± 0.08 ^d^	19.53 ± 2.28 ^b^	25.01 ± 2.26 ^c^
**20**	5.16 ± 2.91 ^c^	2.87 ± 0.29 ^d^	17.29 ± 2.97 ^b^	22.81 ± 3.07 ^c^

DW: dry weight; FW: fresh weight. ^a–d^ indicate the SCG treatments that have statistically significant differences (*p* < 0.05) from the given mean.

Crop success depends on nutrient input and incorporation, particularly nitrogen. Therefore, the organic nitrogen in the plants was evaluated, as reported in Table 2. Total carotenoids content, including neoxanthin, violaxanthin, lactucaxanthin, lutein and β-carotene, is also given in Table 2, side by side with total chlorophylls (Chl A, Chl B and pheophytins), and expressed as mg/100 g fresh weight, for a global discussion of the spent coffee effects.

The results clearly demonstrate that nitrogen amounts are higher in control samples, and decrease proportionally to the edible plant biomass, in opposition to SCG increase. Therefore, the slightly higher biomass in the 2.5% and 5% groups is not supported by a nitrogen increase. For higher SCG amounts the results are clearer, with an effective reduction in both weight and nitrogen content. Still, total carotenoids and chlorophylls follow the pattern observed for each compound individually, with a clear enhancement of their content per 100 g fresh weight.

According to Bumgarner and co-workers [18], plant tissue N levels tend to increase with N application in soil. Once SCG are characterized by high nitrogen content (Table 3) an increased yield could be expected. However, as SCG amounts in soil increase, and therefore apparently N supply, lettuce tissue N levels progressively declined for all the SCG amounts tested (Table 2). SCG nitrogen is mainly attributed to organic compounds, including proteins, melanoidins, and caffeine, among others, while mineralized nitrogen is taken by plants roots, as (NO^3−^) and ammonium (NH_4_^+^). Several authors have demonstrated that after incorporating compost and manure, net immobilization in the first crop season can occur followed by mineralization during the second crop [19]. Also, it is necessary to consider that availability of nutrients in organic fertilizers does not depend on its total content in the material but on the dynamics of the process as some elements can become more available depending on pH, moisture, aeration, *etc*. Both vegetable soil and SCG added revealed a pH within recommend, usually around 6.0 for most vegetables, including lettuce [20]. Even though SCG is generally recognized as an acid compounds-rich material, with high amounts of organic and chlorogenic acids, among others [1], pH was not significantly reduced.

**Table 3:** Physicochemical characterization of vegetable soil and SCG used for cultivation.

**Parameters**	**Soil**	**SCG**
Moisture (%)	55	63
Apparent density (g.L^−1^)	311	600
pH (1:5 H_2_O)	6.0	5.6
EC (mS.cm^−1^)	1.2	1.5
Total N (%)	0.3 *	n.d.
Organic N (%)	n.d.	1.2
P (%)	0.02	0.02
K (%)	0.31	0.35
Mg (%)	0.13	0.10
Caffeine (%)	n.d.	0.18

n.d.: not determined; * as reported on the label.

The previous observations support the assumption that plants were not accumulating adequate amounts of nitrogen. The main symptom of N deﬁciency in plants is leaf senescence caused by lipid peroxidation and pigment loss, specially carotenoids and chlorophylls, as well as protein degradation, both leading to the inhibition of photosynthetic capacity [21]. Nevertheless, the pigments concentration in the present study increased when leaf N amounts decreased. The carotenoids and chlorophylls amounts are known to provide information about the level of stress the plant is experiencing [22]. Kim and co-workers [15] reported that the total amount of carotenoids in lettuce increases when plants are subjected to nutritional stress. Probably, once some carotenoids have strong antioxidant capacity, they grant protection against photo-bleaching of chlorophyll [8].

It should be highlighted that SCG themselves cannot be regarded as a simple vegetable compound. The SCG used under the present study were obtained after the preparation of espresso coffee, being rich in several bioactive compounds (Table 3), namely caffeine, with 180 mg per 100 g of SCG. Caffeine is a natural secondary metabolite with biological roles in some plants, as coffee and tea, serving as a chemical defence mechanism. However, when other plants are expose to caffeine, adverse effects have been reported. A mitotic delay and potentiation of chromosome damage has been reported in proliferating plant cells, retardation in seedling growth, and induction of early plant senescence [23]. These authors studied caffeine effect in seedling growth, with documented modification at 1mM, falling within the concentration used under the present study. One can suppose that the increased carotenoids and chlorophyll contents might be regarded as internal signalling for potential toxicity or at least stress, likely associated with caffeine. Further studies will be undertaken to explore this phenomena.

## 3. Experimental

### 3.1. Spent Coffee Grounds

Fresh spent coffee grounds were collected from several coffee establishments serving espresso coffee on a regular basis. A total of 30 kg was obtained, homogenized in a 100 L conical mixer, and sampled for physical and chemical characterization. Based on the spent coffee apparent density (600 g·L^−1^) and that of the standard vegetable soil (Siro® Germe, Leal e Soares, Lda.; Portugal) used for dilution (Table 3), five mixtures were prepared: 2.5%; 5%, 10%, 15% and 20%, all on a volume basis, using plain vegetable soil as control (0%). A total of 50 kg were prepared for each mixture, adequately homogenized, and distributed by 1L plastic pots.

### 3.2. Plant Material and Growth Conditions

All experiments were conducted in the greenhouses of the Agrarian School of the Polytechnic Institute of Bragança, (Portugal, 41° 47' 47.50918" N, 6° 46' 5.71990" W), with adequate ventilation, temperature controlled at 25 ± 2 °C, natural light, and covered with a transparent plastic film to achieve adequate light amounts and enhanced photosynthesis. Lettuce seedlings (*Lactuca sativa* L. var. *capitata* cv. “Four Seasons”) were pre-grown in expanded polystyrene containers (294 cavities, each with a 15 cm^3^ capacity) filled with organic substrate for germination (Siro® Germe, Leal e Soares, Lda.; Portugal) (February 2011). After 32 days, the healthy plants were transferred to the 1L plastic pots containing mixtures with different percentages of fresh SCG, being labelled as follows: 0% (control), 2.5%, 5%, 10%, 15% and 20% (v/v). For each SCG percentage, 21 pots were prepared. All pots were irrigated with 50 mL of water immediately after transplanting, and distributed randomly within the greenhouse longitudinal extension. During the growing period, corresponding to the spring season in the Northern hemisphere, all plants were irrigated with 50 mL of water (every 2 days). In two of these irrigation periods (at the 7th and 21th days), 0.2% (v/v) of Complesal 12-4-6 (N/P/K) nutritive solution (Bayer, Portugal) was added. Some pots were eliminated during this period, due to diversified problems, finishing with 15 plants per treatment, on average.

Lettuce plants were harvested after 39 days. After careful wash of the healthy plants with deionized water, the roots were separated from the edible part and their weight recorded. All samples were placed in polyethylene bags and immediately frozen at −18 °C. Due to their reduced individual weight, three plants were frozen in the same bag, being thereafter treated as one sample and named as a group (Table 1). Adequate care was taken to maintain the samples at the same temperature during their transportation to the laboratory were the chemical analyses were performed, being again immediately conditioned at −18 °C. Despite several studies resort to lyophilization for sample preservation [24,25], for carotenoid analysis this process results in signiﬁcant degradation of β-carotene as it increases sample porosity, and consequently exposure to oxygen during storage [10]. Therefore, immediately before analysis, the plants were defrosted, homogenized in a food processor (Silvercrest, Germany), and immediately sampled for the chemical analyses. Knowing that carotenoids are not homogeneously distributed in the vegetable tissues, particularly within lettuce leaves, adequate sample homogenization was of extreme importance. Subsamples were also taken for moisture content evaluation, by oven drying at 103 °C (WTC Binder; Germany).

### 3.3. Carotenoid and Chlorophyll Extraction

Carotenoids are extremely unstable, requiring special measures to prevent their degradation during extraction [10]. These inconveniences were partially controlled by the addition of antioxidants (butylated hydroxytoluene, BHT), use of acid-free brown glassware, sunlight exclusion from our laboratory and short-time and low temperature operating conditions. Still, some conversion of chlorophylls into pheophytins was observed, which could have benefitted from pH control. Since lettuce is a low-lipid material and essentially free from carotenoid esters [10], saponification was not included.

Extraction of carotenoids was performed according to the method reported by Zhou *et al.* [26] with some modifications. After being defrosted and homogenized, triplicate amounts of lettuce samples (around 500 mg each) were macerated for 1 h, under stirring, with 5 mL of cold chloroform:methanol (2:1, v/v), containing BHT (0.1%; w/v) (Sigma; Germany) and *trans*-β-apo-8′-carotenal (Fluka, Germany) as internal standard (IS). The chloroform layer was separated by centrifugation (5,000 rpm for 3 min) after adding NaCl aqueous solution 1 mL of (0.9%; w/v) aqueous solution. This procedure was repeated twice and the three organic extracts were combined, concentrated under a gentle nitrogen stream and recovered with a total volume of 1 mL of ethyl acetate. After a further centrifugation at 13,000 rpm (5 min), the supernatant was separated and kept refrigerated until the HPLC analysis, within the same working day. 

### 3.4. HPLC Separation and Quantification

The HPLC system was from Gilson (France), consisting of two pumps (305 and 306), an 805 manometric module, a 811C dynamic mixer, an injection port with a 20 µL loop (Rheodyne, USA) and a photodiode array detector (Varian Prostar, USA) controlled by a data processor software (Varian Star Workstation, USA). 

HPLC analysis was carried out according to the procedure describe by Caldwell and Britz [24], with minor adjustments. For analysis of lettuce extracts, 20 µL of the extract was injected, in duplicate, into a 250 × 4.6 mm, Phenomenex Luna ODS-2 (octadecylsilyl) (5 μm particle size) column (USA) and eluted with a 30 min. linear gradient from 20% ethyl acetate (Sigma-Aldrich, Germany) and 80% aqueous methanol (v/v; Merck, Germany) to 100% ethyl acetate, always with 0.05% (v/v) triethylamine (Alfa-Aesar, Germany). The flow rate was 1 mL·min^−1^ with the temperature maintained at 25 °C. The elution in 100% ethyl acetate continued for 5 min. before returning to the starting conditions and the column was equilibrated for 5 min. before the next sample injection.

The chromatograms were acquired from 190 to 650 nm. For identification purposes, spectra were analysed at the 350–550 nm band and compared with authentic standards (lutein, β-carotene, chlorophyll *a* and pheophythin *a*) or literature data [13]. All compounds were quantified on the basis of the chromatograms extracted at 440 nm, except for chlorophyll *a* and pheophythin *a*, quantified at 412 nm.

Quantitative analysis was carried out by the internal standard method, using calibration curves, assembled with a minimum of six concentrations for each standard and subjected to the entire extraction procedure. The authentic standard *trans*-β-carotene, and the IS were purchased from Fluka Chemie, while lutein and chlorophyll *a* were obtained from Sigma-Aldrich, and pheophytin *a* obtained from acidified chlorophyll *a* solution. The concentration of the individual standards was previously evaluated by UV/VIS spectrophotometry, using published absorption coefficients [13]. The linear portions of the standard curves were used to convert the integrated areas to mg pigment/100 g fresh weight. Lutein calibration curve was used for neoxanthin, violaxanthin and lactucaxanthin quantification, while Chl A recorded at 440 nm was used for Chl B estimation. Pheophytin *a* was present in variable amounts, being calculated and reported as Chl A.

The analytical method was validated under our laboratorial conditions, with adequate performance for the purposes of this study. As detailed in Table 4 an adequate linear working range was obtained. The chromatographic system was highly stable, with small retention time variations. Based on triplicate analysis of a randomized sample, coefficients of variation (CV%) inferior to 4% were obtained for the compounds under study.

**Table 4:** Analytical method performance.

	Lutein (440 nm)	β-Carotene (440 nm)	Chlorophyll *a *(412 nm)
Working range (μg/mL)	1.4–21.2	1.8–27.8	1.9–58.0
R^2^	0.9995	0.9860	0.9965
Retention time (min)	11.96 ± 0.10	24.54 ± 0.07	22.27 ± 0.18
Precision (CV%)	3%	2%	4%

### 3.4. Other Chemical Analyses

Spent coffee grounds and vegetable soil were analysed for pH and electrical conductivity in aqueous extract (1:5 w/v). Organic nitrogen was evaluated by the Kjeldhal method, total Mg by flame photometry, total K by atomic absorption spectrometry, and total P by spectrophotometry, using standard protocols. Caffeine was evaluated by HPLC, after hot water extraction, and quantified by external standard.

### 3.5. Statistical Analyses

Statistical analyses were performed using GraphPad Prism 5.04 (GraphPad Software, Inc.; USA). Differences of the photosynthetic compounds with SCG content were calculated using one way analysis of variance (ANOVA) with Tukey’s post-hoc procedure, with a level of significance at *p* < 0.05. To ensure that data came from a normal distribution, standardized Skewness and standardized Kurtosis were verified.

## 4. Conclusions

The present study shows that the incorporation of reduced amounts of fresh SCG (up to 10% v/v) in the cultivation media can increase the amounts of xanthophylls, β-carotene and chlorophylls in lettuce leaves, together with a slight increase in their biomass. This effect is particularly interesting from a nutritional point of view. A simultaneous decrease was observed in the amounts of N in the leaves, for all the SCG groups, indicating that the plant was probably under nutritive stress or that some SCG compounds, probably caffeine, induces some toxicity, activating a signalling stress in plant, probably associated with the increased carotenoids content. Further studies are therefore required in order to elucidate the effectiveness of this practice, regarding both lettuce nutritional qualities and induced stress.
